# Maternal care quality in near miss and maternal mortality in an academic public tertiary hospital in Yogyakarta, Indonesia: a retrospective cohort study

**DOI:** 10.1186/s12884-017-1326-4

**Published:** 2017-05-22

**Authors:** Yuli Mawarti, Adi Utarini, Mohammad Hakimi

**Affiliations:** 1 Public Health Graduate Program, Faculty of Medicine, Universitas Gadjah Mada, Sekip, Yogyakarta, 55281 Indonesia; 2 Department of Public Health, Faculty of Medicine, Universitas Gadjah Mada, Sekip, Yogyakarta, 55281 Indonesia; 3 Department of Obstetrics and Gynaecology, Faculty of Medicine, Universitas Gadjah Mada, Sekip, Yogyakarta, 55281 Indonesia

**Keywords:** Quality of maternal care, Process indicators, Response time, Timeliness, Maternal near miss, Maternal mortality, Academic hospital

## Abstract

**Background:**

Reducing maternal mortality remains a major challenge for health care systems worldwide. The factors related to maternal mortality were extensively researched, and maternal death clusters around labour, delivery and the immediate postpartum period. Studies on the quality of maternal care in academic medical centre settings in low income countries are uncommon.

**Methods:**

A retrospective cohort study of maternal deaths was conducted in an academic public tertiary hospital in Yogyakarta, and maternal near misses were used as controls. Data were obtained from medical records from February 1, 2011 to September 30, 2012. Three groups of variables were measured: (1) timeliness of care, (2) adherence to a standard of process indicators, and (3) associated extraneous variables. Variables were analysed using logistic regression to explore their effects on maternal mortality.

**Results:**

The mean of triage response time and obstetric resident response time were longer in maternal deaths (8 ± 3.59 and 36.17 ± 23.48 min respectively) compared to near misses (1.29 ± 0.24 and 18.78 ± 4.85 min respectively). Near misses more frequently received oxytocin treatment than the maternal deaths (OR 0.13; 95%CI 0.02–0.77). Magnesium sulfate treatment in severe-preeclampsia or eclampsia was less given in maternal deaths although insignificant statistically (OR 0.19; 95% CI 0.03–1.47). Prophylactic antibiotic was also more frequently given in near misses than in maternal deaths though insignificant statistically (OR 0.3; 95% CI 0.06–1.56). Extraneous variables, such as caesarean sections were less performed in maternal deaths (OR 0.15; 95% CI 0.04–0.51), vaginal deliveries were more frequent in maternal deaths (OR 3.47; 95% CI 1.05–11.54), and more women in near misses were referred from other health care facilities (OR 0.09; 95% CI 0.01–0.91).

**Conclusions:**

The near misses had relatively received better quality of care compared to the maternal deaths. The near misses had received faster response time and better treatments. Timely referral systems enabled benefits to prevent maternal death.

## Background

Reducing maternal mortality remains a major challenge for health care systems worldwide. Only 34 out of 75 priority countries, where 95% of global maternal and children deaths occurred, had achieved MDG 4 and 5 [1]. Despite the global challenges, most countries claimed high coverage of antenatal care (ANC) and deliveries by health workers [2, 3].

This situation is also present in Indonesia. Indonesia did not achieve MDG 5, i.e. average annual rate of reduction was 3.5% (1990–2013) and maternal mortality ratio was 190/100,000 live births in 2013 [1]. The coverage for at least one visit (ANC + 1) and at least four visits (ANC + 4) in Indonesia were 93 and 82%, respectively, during 2005–2012 and the coverage of births attended by health workers was 80% [3, 4]. Maternal death clusters around labour, delivery and the immediate postpartum period and reflects the strength of the health care system [5, 6]. Health facility capacity in providing emergency obstetric care is critical to avert maternal complications and adverse events [7, 8]. A study performed in three Colombian hospitals claimed that 71% of adverse events were avoidable [9]. Maternal deaths occurred in a hospital can be used to asses its maternal care quality [10]. A recent multicentre cross-sectional study in a Brazilian network found that delays related to the quality of medical care/sub-standard care were positively associated with severe maternal outcome [11, 12]. A cross-sectional study in Banten, Indonesia, demonstrated that the prevalence of near misses was much greater in public hospitals than private hospitals [13].

Investigation on the causes, pattern and timing of maternal near miss and mortality provides information to improve the quality of care [14]. Research in factors associated with maternal mortality was extensively performed, but data on the role of delays in providing care in health care facilities are sparse [15, 16]. The present study was conducted to identify maternal care quality provided to mothers with life threatening conditions in an academic public tertiary hospital and the relationship of quality of care with maternal mortality in Yogyakarta.

## Methods

### Setting

The study was conducted in an academic public tertiary hospital in Yogyakarta, namely, the Dr. Sardjito hospital. This hospital has 750 beds, and it is designated as a referral centre in the Yogyakarta province and the southern part of the Central Java province. The hospital performs approximately 2000 deliveries and 700 obstetric surgeries annually. Approximately 3.5 million people inhabit the Yogyakarta Special Region, and health care facilities are delivered at 320 auxiliary health centres, 121 primary health care centres, and 66 hospitals [17, 18]. Skilled health workers attended most of the 45,900 births, and ANC coverage was 100% for both at least one visit and at least four visits [19–21].

### Study population

The study population was all women who gave birth in the Dr. Sardjito hospital during the study period (February 2011–September 2012).

### Design

A retrospective cohort study was performed. Screening was conducted in hospital electronic data, forensic department and nurse records to find cases that meet inclusion criteria adopted from WHO near miss approach (2011). Women are pregnant, in labour or who delivered, or aborted up to 42 days ago arriving at the hospital with any of the listed conditions or those who develop any conditions mentioned in the inclusion criteria of WHO near miss approach during their stay at the hospital would be eligible [15].

The outcome in this study was quality of care, measured by identifying pregnancy outcome (maternal death and near miss) during or at the end of hospitalization. Variables used as predictors to measure the quality of care were categorised into 3 groups: (1) timeliness indicators, (2) the process indicators of care based on WHO near miss approach, and (3) extraneous variables.

Timeliness indicators consisted of triage response time, obstetrician resident response time, intensive care unit (ICU) waiting time, and length of stay in the emergency room. Response time (minute) was defined as the time needed by triage personnel to check the patient at arrival in the emergency room (continuous). Obstetrician response time (minute) was defined as the time taken by an obstetric resident to examine the patient for the first time after triage referred the patient to the obstetric department (continuous). The Intensive Care Unit (ICU) waiting time (minute) was defined as the period of time since the decision of the ICU care team was made until the patient arrived in the ICU (continuous). Length of stay in the emergency room (minute) was defined as the period of time spent in the emergency department before transfer to other parts of hospital (continuous). Process indicators comprised treatment with oxytocin, treatment with magnesium sulfate for preeclampsia or eclampsia, antibiotic treatment for infection or sepsis and antibiotic prophylactics for infection. Measured extraneous variables consisted of referral status (nominal), hypertension and asthma as co-existed comorbidities (nominal), and mode of delivery, vaginal delivery or caesarean section (nominal).

### Data collection

The electronic medical records identified 318 eligible mothers out of the 3300 deliveries during the study period. A total of 114 mothers were enrolled in this study: 29 cases of maternal deaths, with one inaccessible medical record, and 86 near misses as controls. The other 203 potential subjects were not enrolled into the study because their physical medical records were unavailable at the time of study. A logbook was used to keep subjects confidential information, including the subject’s identity and medical records number. Data collected from physical medical records consisted of subjects’ characteristics and disease history, referral status, response time, vital sign, diagnoses, laboratory test results, treatment and intervention given, length of stay and pregnancy outcome.

### Data analysis

Timeliness, process indicators and extraneous variables of maternal care quality are presented as percentages and analysed using logistic regression. Odds ratio (OR) and confidence intervals (CI) obtained for all maternal care quality associated variables. Some variables with OR 1, were omitted from logistic regressions, including antibiotic treatment and heart disease as comorbidity. A multi-predictors logistic regression also was performed to measure interaction effect between predictors of timeliness variable.

## Results

Table 1 shows that the maternal death and near miss groups had high ANC coverage (87.5 and 97.2%, respectively). The maternal death group compared to the near miss group had a shorter mean gestational age (33.11 ± 1.88 and 35.5 ± .77 weeks, respectively), smaller estimated foetal weight (1876.5 ± 287 and 2119.4 ± 191.50 g, respectively) and a higher percentage of stillbirths (41.67 and 5.95%, respectively). History of Preeclampsia/eclampsia in previous pregnancy was higher in maternal death (16.7%) than the near miss (5.95%). History of postpartum haemorrhage was only found in the near miss (3.53%).

**Table 1:** Characteristics of mothers who died and near misses in the Sardjito hospital during the study period (*n* 114)

Characteristics	Maternal Death (28)	Near Miss (86)
Age (mean ± standard deviation)	31.64 ± 2.40	30.19 ± 1.67
Gestation age in current pregnancy (weeks)	33.11 ± 1.88	35.5 ± 0.77
Estimated Foetal weight at admission (gram)	1876.5 ± 287	2119.4 ± 191.50
Parity (*n*, %):		
Primigravid	8 (30.77%)	43 (50.0%)
Secundi or multigravida	16 (69.23%)	43 (50.05%)
Referral from other health care facilities (*n*, %)	23 (88.46%)	85 (98.84%)
Diagnoses during hospitalization (*n*, %):		
Eclampsia	6 (21.43%)	24 (27.91%)
Severe Pre-eclampsia	17 (60.7%)	82 (95.35%)
HELLP or partial HELLP syndrome	7 (25%)	28 (32.56%)
Sepsis or severe systemic infection	17 (18.42%)	4 (4.65%)
Postpartum Haemorrhage	4 (14.29%)	5 (5.81%)
Ruptured uterus	0 (0.00%)	1 (1.16%)
Severe complication of abortion	1 (3.57%)	0 (0.00%)
Admission to intensive care unit (n,%)	24 (85.71%)	39 (45.35%)
Use of blood products (*n*, %)	6 (24%)	19 (22.89%)
Stillbirth in current pregnancy (*n*, %)	10 (41.67%)	5 (5.95%)
ANC history in current pregnancy (*n*, %)	14 (87.50%)	72 (97.20%)
Timeliness (mean ± standard deviation):		
Triage response time (minute)	8 ± 3.59	1.29 ± 0.24
Obstetric resident response time (minute)	36.17 ± 23.48	18.78 ± 4.85
ICU waiting time (minute)	109.5 ± 61.4	73.8 ± 19.35
Surgery waiting time (minute)	119.7 ± 34.75	142.5 ± 23.15
Length of stay in ICU (hour)	143.5 ± 87.21	56.16 ± 23.58
Length of stay in emergency (minute)	252.05 ± 90.66	202.28 ± 35.34
Process Indicators (*n*, %):		
Oxytocin treatment	22 (84.62%)	83 (97.65%)
Magnesium sulphate in Preeclampsia/Eclampsia	15 (88.24%)	78 (97.50%)
Antibiotic as prophylactic	24 (88.90%)	81 (96.43%)
Antibiotic as treatment	25 (92.69%)	84 (100,00%)
Comorbidities (*n*, %):		
Hypertension	6 (25.00%)	14 (16.47%)
Diabetes Mellitus	0 (0.00%)	1 (1.19%)
Heart Disease	6 (26.10%)	0 (0.00%)
Asthma	3 (12.50%)	3 (3.53%)
Infectious disease	3 (13.0%)	0 (0.00%)
Mode of delivery:		
(Emergency) Caesarean section (*n*, %)	19 (70.37%)	80 (94.12%)
Vaginal delivery (*n*, %)	6 (24.00%)	7 (8.33%)
Induction in Labour (*n*, %)	5 (19.23%)	13 (15.66%)
Hysterectomy (*n*, %)	3 (11.11%)	3 (3.57%)
History of Preeclampsia/Eclampsia in previous pregnancy (*n*, %)	4 (16.67%)	5 (5.95%)
History of Postpartum haemorrhage in previous pregnancy (*n*, %)	0 (0.00%)	3 (3.53%)

### Timeliness and maternal outcome

Timeliness consisted of triage response time, obstetric resident response time, ICU waiting time and surgery waiting time. Table 1 shows that each predictor of timeliness was longer in maternal death as compared to the near miss, except for the surgery waiting time. Table 2 demonstrates that triage response time exhibited the highest OR compared to the other timeliness components (OR 1.88; 95% CI 1.35–2.62). Obstetric resident response time (OR 1.02; 95% CI 1.00–1.04), surgery waiting time (OR 1.00; 95% CI 1.00–1.01) and ICU waiting time (OR 1.01; 95% CI 1.00–1.01) were insignificantly different between the maternal death and near miss groups.

**Table 2:** Timeliness in predicting maternal death in the Sardjito hospital, in single and multi-predictors models

Predictors	OR 95% CI *p*						
	Model 1	Model 2	Model 3	Model 4	Model 5	Model 6	Model 7
Triage response time	1.88 1.35–2.62 <0.001	-	-	-	2.01 1.24–3.27 0.005	1.83 1.33–2.94 0.013	3.82 0.25–58.94 0.337
Obstetric resident response time	-	1.02 1.00–1.04 0.039	-	-	1.01 0.96–1.06 0.784	-	0.98 0.877–1.11 0.773
ICU waiting time	-	-	1.01 1.00–1.01 0.18	-	-	1.01 0.98–1.02 0.762	0.86 0.64–1.14 0.284
Surgery waiting time	-	-	-	1.00 1.00–1.01 0.239	-	-	-
−2 Log likelihood	32.78	65.66	72.75	83.75	20.90	15.21	7.67

### Process indicators of care and maternal care outcome

Table 1 and 3 show that there is a trend for all interventions to favour the near miss cases, but the trend is only statistically significant for oxytocine (OR 0.13; 95% CI 0.02–0.77). Treatment with magnesium sulfate in severe preeclampsia/eclampsia was more frequent in the near miss cases (97.50%) than maternal death (88.24%), but the difference is not statistically significant (OR 0.19; 95% CI 0.03–1.47). Intervention of antibiotic as prophylaxis was not significantly higher in the near miss cases than in maternal death (96.43% vs 88.90%; OR 0.30; 95%CI 0.06–1.56). All near miss cases received antibiotic as treatment and only 92.69% of maternal death received it.

**Table 3:** The process of care indicators in predicting maternal death in the Sardjito hospital

Independent variables	OR	95% CI	*p*
Oxytocin treatment (n 109)	0.13	0.02–0.77	0.025
Magnesium Sulfate treatment (n 97)	0.19	0.03–1.47	0.113
Prophylactic Antibiotic (n 111)	0.3	0.06–1.56	0.152

### Extraneous variables and maternal outcome

Table 1 shows that the maternal death group exhibited a higher percentage of comorbidity compared to the near miss group, including hypertension (25 and 16.47%, respectively). The respective figures for other comorbidities in the maternal death group and the near miss group were 26.1 and 0% for heart disease, 12.5 and 3.53% for asthma, and 13 and 0% for infectious disease.

Most extraneous variables in this study were exhibited no differences between the death and near miss groups, except referral status and cesarean section (Table 4). The survived mothers were more likely to be referred from other health care facilities (OR 0.09; 95% CI 0.01–0.91). Caesarean delivery in this study was an emergency intervention, and both groups exhibited a high percentage of caesarean sections as the final mode of delivery (70.37 and 94.12% in maternal death and near miss groups, respectively). Caesarean section also showed significant difference in the maternal death and near miss groups (OR 0.15; 95% CI 0.04–0.51). The maternal death group was more likely to undergo vaginal delivery as the final mode of delivery (OR 3.47; 95% CI 1.05–11.54). Among the six mothers who underwent hysterectomy, the mode of delivery comprises spontaneous vaginal delivery (two cases in the near miss and two cases in maternal death), caesarean delivery (one case in maternal death), and vaginal delivery with misoprostol induction (one case in near miss).

**Table 4:** Extraneous variables in predicting maternal death in the Sardjito hospital

Predictors	OR	95% CI	*p*
Referral status (referred from other care provider)	0.09	0.01–0.91	0.041
Hypertension as comorbidity	1.69	0.57–5.01	0.334
Asthma as comorbidity	1.69	0.74–20.75	0.11
Caesarean section	0.15	0.04–0.51	0.002
Vaginal delivery	3.47	1.05–11.54	0.042

## Discussion

This study was a case–control study of mothers with life threatening conditions who attended tertiary academic hospitals in a low middle income country setting. The study compared maternal care quality between the maternal death and near miss groups and found that improving the quality of care in life threatening maternal cases in a designated referral tertiary-care hospital was important in averting maternal death. This study highlighted, for the first time, the need to improve timeliness in general. Response time in providing immediate treatment to mothers with life threatening conditions must be improved in a country such as Indonesia, which has exhibited slow progress in decreasing the maternal mortality ratio.

Only 114 out of 318 mothers with life threatening conditions were enrolled in this study because of the medical record of the remain (204) were inavailable in the department of medical records during the study period, which suggesting poor implementation of medical record system. Failure of the hospital record retention system could affect the quality of care [22, 23]. In 2011, the hospital electronic medical record system had not been established, and medical records were paper-based. Problem in completeness and delays in returning medical records were common.

Twenty-eight of the 114 mothers with life threatening conditions experienced death and had worse outcomes of pregnancy. Stillbirth was common in the maternal death and near miss groups, but the percentage was higher in the maternal death group. Gestational age was shorter in the maternal death group as compared to the near misses.

Timeliness is an important dimension of quality of care [24]. This study found that delays in the initiation of care occurred in all stages of care, including triage response time, obstetric resident response time, and ICU waiting time. Long delays occurred in all measures of timeliness, but the maternal death group had significantly longer triage response times compared to the near miss group. Delays in triage response may be followed by further delays in providing treatment. However, delays may also occur as a result of a shortage of hospital resources in the management of severe maternal cases. Twenty-four-hour attending physicians and nurses are very important in the setting where EmOC is provided [25]. Timely access to providers is a critical dimension of emergency care quality performance [26]. Longer waiting times jeopardize the patient’s health and lead to worse outcome [27–30]. Triage response time was the most efficient variable for predicting the outcome of maternal cases with life threatening conditions. Life-saving actions must be performed immediately with a response time less than 1 min because the success of resuscitation decreases approximately 7–10% per minute [31]. Cardiac arrest or deterioration may develop anytime in women with life threatening conditions. Witnessed cardiac arrests have a better chance for timely treatment [32]. Successful resuscitation following cardiac arrest requires an integrated set of coordinated actions between health workers [33]. Hospitals must systematically address problems in time delays to improve quality of care. Longer waiting times also provoke dissatisfaction, which is another quality of care dimension, in addition to increased length of stay and mortality risk [34].

The quality of care investigated in this study also focused on care processes according to WHO near miss approach, i.e., treatment with oxytocin, magnesium sulfate for severe preeclampsia/eclampsia, antibiotics as prophylactics and antibiotic treatment when required [35–37]. WHO recommends oxytocin administration, and this study found that the near miss group was more likely to receive oxytocin. Oxytocin administration exerted a protective effect. Magnesium sulfate treatment for severe preeclampsia/eclampsia is slightly more frequent in the near miss group, however, the difference is not statistically significant. This result may indicate the association of other dominating factors with pregnancy outcome. WHO recommends magnesium sulfate administration in severe preeclampsia to prevent seizures and in eclampsia as an anticonvulsant [38]. Predicting who has an eclamptic seizure risk is difficult, but magnesium sulfate administration is necessary in severe-preeclampsia/eclampsia cases [39]. Antibiotics for infection is one recommended process indicator of maternal care [15]. Infection prevention and treatment are important to prevent mothers with life threatening conditions from developing sepsis and undergoing longer hospitalization [40]. No significant difference in antibiotic administration as treatment or prophylaxis on the outcome of pregnancies with life threatening conditions was demonstrated in this research, but the near miss group had a higher percentage of antibiotic administration than the maternal death group.

Preeclampsia/eclampsia history was more common in the maternal death group compared to the near miss group. However, history of post-partum haemorrhage was only found in the near miss group, and statistically unable to explain its effect on pregnancy outcome.

Mothers referred from other health care facilities were more likely to have a better outcome, which may indicate a potential benefit of timely referral system to avert maternal mortality. High ANC coverage and the existence of the referral system should benefit mothers and minimize delays in household decision making (first delay) and accessing emergency care services (second delay). This study highlights the urgency to improve the quality of obstetric emergency care in tertiary academic hospitals. Delays occurred when health care providers failed to deliver qualified treatment as required [41].

Comorbidities, such as hypertension, heart diseases, asthma and infectious diseases, existed in the maternal death and near miss groups, but a higher percentage was found in the maternal death group. Therefore, this should have raised an early awareness in the frontline caregiver to promptly refer the mothers.

The study limitations were related to the unavailability of medical records. However only one maternal death occurred among those without physical medical records. This study was conducted in tertiary teaching hospital, in an area with good access to primary health care centers and referral hospitals. Thus, generalisability of the study would depend on similarities to this local context.

## Conclusions

Near miss mothers who accepted a higher quality of care from health care facilities have a better chance of achieving better outcomes compared to mothers who died. The near miss group received a better response time, particularly the triage response time. Oxytocin administration was the only process indicator that provided a protective effect to the outcome. Mothers who were timely referred had better outcome. Overall, surviving mothers with life threatening conditions received a higher quality of care compared to mothers who died. The quality of maternal care services, particularly in timeliness and standardized care, must be improved.

## Abbreviations

CI: Confidence interval
EmOC: Emergency obstetric care
ER: Emergency room
HELLP syndrome: Hemolysis, elevated liver enzymes levels and low platelet count syndrome
ICU: Intensive care unit
MMR: Maternal mortality ratio
OR: Odds ratio
